# What Matters in Weight Loss? An In-Depth Analysis of Self-Monitoring

**DOI:** 10.2196/jmir.7457

**Published:** 2017-05-12

**Authors:** Stefanie Lynn Painter, Rezwan Ahmed, James O Hill, Robert F Kushner, Richard Lindquist, Scott Brunning, Amy Margulies

**Affiliations:** ^1^ Retrofit INC Chicago, IL United States; ^2^ Retrofit, INC Chicago, IL United States; ^3^ University of Colorado Aurora, CO United States; ^4^ Northwestern University Feinberg School of Medicine Chicago, IL United States; ^5^ Swedish Medical Center Seattle, WA United States

**Keywords:** behavior, body mass index, fitness trackers, self-monitoring, obesity, overweight, weight loss

## Abstract

**Background:**

Using technology to self-monitor body weight, dietary intake, and physical activity is a common practice used by consumers and health companies to increase awareness of current and desired behaviors in weight loss. Understanding how to best use the information gathered by these relatively new methods needs to be further explored.

**Objective:**

The purpose of this study was to analyze the contribution of self-monitoring to weight loss in participants in a 6-month commercial weight-loss intervention administered by Retrofit and to specifically identify the significant contributors to weight loss that are associated with behavior and outcomes.

**Methods:**

A retrospective analysis was performed using 2113 participants enrolled from 2011 to 2015 in a Retrofit weight-loss program. Participants were males and females aged 18 years or older with a starting body mass index of ≥25 kg/m2, who also provided a weight measurement at the sixth month of the program. Multiple regression analysis was performed using all measures of self-monitoring behaviors involving weight measurements, dietary intake, and physical activity to predict weight loss at 6 months. Each significant predictor was analyzed in depth to reveal the impact on outcome.

**Results:**

Participants in the Retrofit Program lost a mean –5.58% (SE 0.12) of their baseline weight with 51.87% (1096/2113) of participants losing at least 5% of their baseline weight. Multiple regression model (R^2^=.197, *P*<0.001) identified the following measures as significant predictors of weight loss at 6 months: number of weigh-ins per week (*P*<.001), number of steps per day (*P*=.02), highly active minutes per week (*P*<.001), number of food log days per week (*P*<.001), and the percentage of weeks with five or more food logs (*P*<.001). Weighing in at least three times per week, having a minimum of 60 highly active minutes per week, food logging at least three days per week, and having 64% (16.6/26) or more weeks with at least five food logs were associated with clinically significant weight loss for both male and female participants.

**Conclusions:**

The self-monitoring behaviors of self-weigh-in, daily steps, high-intensity activity, and persistent food logging were significant predictors of weight loss during a 6-month intervention.

## Introduction

Self-monitoring is commonly used in weight-loss regimens to increase awareness of current and desired behaviors. Both consumers and health companies are incorporating self-monitoring technology through mobile phone apps, smart scales, and other wearable devices into their weight-loss programs. However, understanding how to best use the information being gathered by this relatively new technology needs more rigorous study, especially with recent controversy regarding the benefits of wearable activity trackers [1,2]. According to the Centers for Disease Control and Prevention (CDC), 36.5% of adults are classified as obese in the United States and US $147 billion is spent on obesity-related medical costs per year; therefore, determining whether and how self-monitoring contributes to weight loss is important for improving the health of the overall population [3].

Standard behavioral treatment in obesity includes dietary and physical activity counseling and self-monitoring of body weight, activity, and diet [4]. Behavioral weight-loss interventions up to 12 months have average outcomes between 5% to 10% weight loss [5-11]. Although clinically significant, the studies reviewed showed less than half of participants are successful at losing 5% or more of their weight [6,7,12-14].

Regular self-weighing or weighing in a consistent pattern over time provides awareness to specific behaviors, situations, or environments that could promote desired or undesired changes in weight. Self-weighing correlates with successful weight loss and has been shown to significantly increase weight loss success in the first 6 months of an intervention [15-18]. Specifically, a minimum of weekly self-weigh-ins has been shown to be effective; however, a higher frequency of self-weigh-ins more than once per week increases weight-loss outcomes [19-24]. Once a consistent pattern of self-weighing has been established, not weighing for more than a month increases likelihood of weight gain, as shown by Helander et al [15].

Both wearing an activity tracker and setting a step goal are associated with lower body mass index (BMI) and an increase in activity [25]. The average American gets 5117 steps per day [26]. High step averages were associated with younger, single males with higher education and lower BMI (kg/m^2^) [26]. Individuals with obesity averaged 1500 fewer steps per day than normal or overweight individuals [26]. Modest weight loss has been shown with pedometer interventions [27,28]. By setting individualized physical activity goals around steps per day and active minutes per day, participants are more likely to increase and maintain physical activity postintervention [29]. More frequent self-monitoring and higher adherence are related to greater physical activity over time, which can lead to a greater decrease in weight at 6 months [30].

Dietary self-monitoring with feedback can improve clinically significant weight-loss outcomes [31-34], whereas personalized feedback can improve consistency of dietary self-monitoring [34,35]. Consistency has the greatest association between dietary self-monitoring and achieving clinically significant weight loss [31-34,36]. Self-monitoring for consecutive days is linked to greater outcomes, such as logging at least one food log entry per day has been shown to increase weight loss [31,32].

The purpose of this study was to analyze the self-monitoring behaviors of participants around weight, activity, and nutrition in a 6-month weight-loss intervention administered by Retrofit (see Multimedia Appendix 1), a personalized weight-management and Web-based disease-prevention solution. The self-monitoring behaviors were evaluated for their association with weight loss to determine the level of impact on predicting weight loss outcomes. Additionally, each high impact behavior was evaluated independently to assess the association between the behavior and weight loss to determine best practices around self-monitoring recommendations. The analysis of the significant self-monitoring behaviors focused on understanding the following questions:

1. What is the association between a participant’s level of self-monitoring and weight loss?
2. What is the association between different levels of weight loss outcomes and the corresponding participant’s commitment to self-monitoring?

## Methods

### Study Design

A retrospective analysis was performed to assess the effect of various self-monitoring behaviors during a 6-month weight-loss intervention using de-identified data from the Retrofit weight-loss program.

### Participants

**Figure 1:**
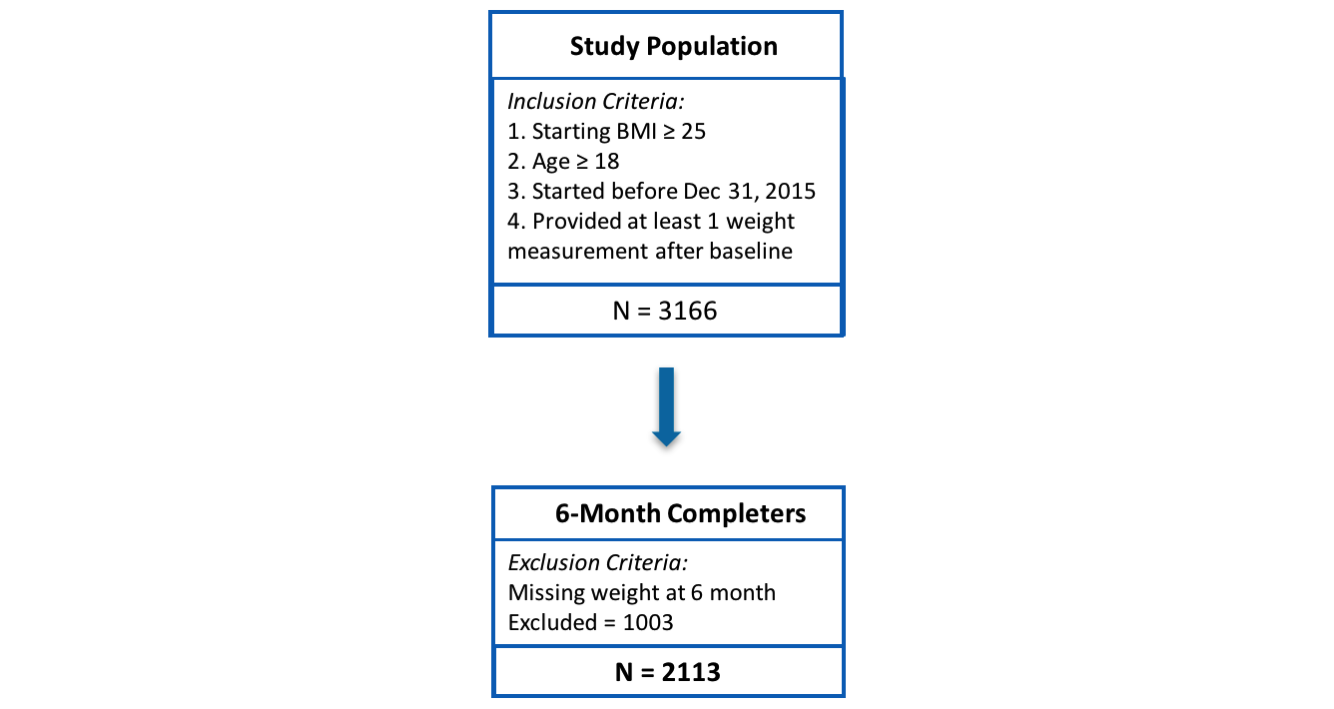
Study population with inclusion / exclusion criteria.

Participants in the study were paying customers of the Retrofit Program who enrolled through the direct-to-consumer website [37] or through an employer-sponsored program. Customers were considered as eligible study participants if they were age at least 18 years; had a starting BMI of 25 kg/m^2^ or higher; had signed up for the program between September 27, 2011 and December 31, 2015; and provided at least one weight measurement beyond baseline measurement. Participants were considered to have completed the program if they provided a weight measurement at the sixth month of their program. A total of 3166 customers satisfied all inclusion criteria to be study participants (Figure 1). Approximately 80.35% (2544/3166) of the study participants were direct-to-consumer customers and the remaining 19.65% (622/3166) were part of an employer-sponsored program. A total of 2113 (66.74%) participants completed the 6-month program. All customers who satisfied the inclusion criteria and provided a weight at 6 months were included as participants. No customer was removed or eliminated from the population due to a lack of success on the program.

### Retrofit Program

The analysis included data from six Retrofit programs: Expert 10 Weight-Loss Program, Expert 15 Weight-Loss Program, Advisor Weight-Loss Program, Jump Start Program, Retrofit Program, and Sustain Program. The Expert 10, Expert 15, Advisor, and Sustain programs were designed with a 6-month weight-loss phase and an additional 6-month weight maintenance phase. The Retrofit Program was designed with a 6-month weight-loss phase only with the option to continue into a maintenance program called Retrofit Next. The Jump Start Program was designed with a 3-month weight-loss phase only with the option to continue into Retrofit Next.

As part of the Retrofit weight-loss protocols, all participants are taught and encouraged to adhere to the same self-monitoring recommendations. All programs provided participants with the same technology, access to a weight-loss expert, education, accountability, feedback, and the opportunity to communicate with an expert coach via Web-based messages. Additional details of the Retrofit Program and expert coach qualifications can be found in a previous publication [38].

The participant was provided a Fitbit activity tracker, Wi-Fi-enabled scale, and access to a private dashboard (see Multimedia Appendix 2). The private dashboard allowed each participant to keep a personal food and exercise log, review his or her personal data, and enabled communication between the participant and his or her expert coach through a Web-based electronic messaging feature (see Multimedia Appendix 3). The private dashboard was accessed via the Retrofitme Web app, mobile website, or mobile phone app, which was available on Apple iOS and Android platforms.

Participants were encouraged to weigh in daily, wear their activity tracker daily, achieve their personalized daily step goal, and log all food and beverage choices consumed throughout the day. Expert coaches personalized participant’s step goals by recommending the participants to increase step counts in increments of 500 to achieve their personal daily step goal at 6 months.

### Measures

#### Weight

Participants were provided a Wi-Fi-enabled scale that securely transmitted weight data over the Internet to a Retrofit central data server. Participant weight data were collected through the use of the provided Wi-Fi scale (99.39%, 556,630/560,043 of recorded weights) or self-reported entry (0.61%, 3413/560,043). Instructions were provided for scale set up, as well as the option for help through Retrofit’s customer support. Self-reported entry was permissible if a participant had difficulty setting up their Wi-Fi scale. Expert coaches reviewed weight data during 1:1 coaching sessions to confirm weight accuracy. Baseline weight was considered as the first weight measurement received from the participant during week 1. Percentage of baseline weight lost at 6 months was calculated and used as the primary outcome. Two weigh-in metrics were calculated to quantify participants’ adherence to self-monitoring behavior and the potential impact of self-weigh-ins on weight loss: (1) the number of weigh-ins per week and (2) the percentage of weeks participants weighed in at least three times.

#### Activity

Participants were encouraged to wear Fitbit activity trackers every day. Activity data from any version of Fitbit activity trackers, such as steps, distance, calories burned, active minutes, etc, were wirelessly uploaded to Fitbit.com and later automatically synced to participants’ personal Retrofit dashboards. Participants did not have the option to self-report activity data. A total of five different metrics were calculated to understand the impact of activity on weight loss. The number of tracker usage days per week was calculated to monitor participant engagement. Step count per day was considered one of the metrics for measuring participant activity. To measure the intensity of the activities, three levels of active minutes were tracked. Fitbit trackers continuously estimate users’ metabolic equivalents (METs) by calculating the intensity of the activity and classifying the active minutes as high, moderate, or low following the CDC’s recommendation [28].

#### Nutrition Tracking / Food Logging

A private online/mobile dashboard allowed participants to track personal food logs. Participants were able to log meals, snacks, treats, and beverages along with the description, quantity, and photo of the food. Each individual meal, snack, treat, and/or beverage was considered a food log entry. Four food logging-specific measures were calculated to quantify participants’ adherence to food logging behavior and the potential impact of food logging on weight loss. The number of days participants logged food entries per week and the number of food log entries per week were calculated to measure the level of adherence to the behavior of food logging. The following two measures were introduced to measure participants’ engagement through food logging over the 6-month intervention: the percentage of weeks participants logged at least five food log entries and at least 15 food log entries.

### Statistical Analysis

All measures associated with self-monitoring behaviors involving weight measurements, dietary intake, and physical activity were included in a multiple regression analysis to predict weight loss during the intervention. Measures with statistically significant contribution to predicting weight loss were identified. To determine self-monitoring behaviors/measures that could be considered as significant predictors of weight loss, three primary regression models were built. The first primary regression model assessed two weigh-in-related measures as predictors of weight loss. The second model included five activity-related measures as predictors of weight loss. The third primary regression model assessed four measures related to food logging as predictors of weight loss. All the significant predictors (ie, self-monitoring behaviors/measures) from the primary regression model were included in an overall regression model that considered all behaviors as predictors of weight loss. The significant predictors of the overall model were considered to be the most important measures/behaviors for weight loss. Finally, each significant self-monitoring measure was analyzed in depth to reveal the impact on outcomes during the intervention period to capture the significant association between high-level monitoring to higher outcome levels. For each behavior, one-way ANOVA tests were performed to determine the association between behavior frequency and weight loss and compare behavior frequency of participants with different weight-loss levels.

Data analyses were performed using R version 3.2.3, which included dplyr 0.4.3, ggplot2 2.1.0, data.table 1.9.6, and leaps 2.9 packages. In addition, *t* tests of equal variance were conducted on continuous variables at baseline and subsequent time points for two group comparisons. One-way ANOVA was utilized to determine mean differences for greater than two group comparisons. Subsequent Tukey tests were conducted to determine mean differences. Chi-square analyses were performed to determine differences among categorical variables when appropriate. To perform best subset selection in a multiple regression analysis, an “all possible regressions” method was used to derive the best-fitting overall model using the leaps package. Alpha was set at .05 for all statistical tests to determine statistical significance.

## Results

The reported results are based on the retrospective analysis evaluating the effect of various self-monitoring behaviors during a weight-loss intervention using 2113 of 3166 participants (66.74%) who completed the Retrofit 6-month weight-loss program.

### Baseline Characteristics

Table 1 shows the demographic details of participants at baseline. There were no differences in age and starting BMI at baseline between male and female participants. Male participants had a higher starting weight (*P*<.001). There were no differences between completers and noncompleters in starting weight (*P*=.07) or starting BMI (*P*=.55), but completers had a higher mean age (mean 44.54, SD 10.72 years vs mean 42.01, SD 10.69 years, *P*<.001; see Multimedia Appendix 4, Table S1).

### Weight Change at 6 Months

The mean weight loss at 6 months was –5.58% (SE 0.12), the mean change in BMI was –1.91 (SE 0.04), and 51.87% (1096/2113) of participants lost 5% or more of their baseline weight (see Table 2). Male participants lost a higher percentage of weight (*P*=.02) and had a higher BMI change (*P*=.01) than female participants. However, there were no significant differences between males and females in terms of the percentage of group losing 5% or more weight at 6 months.

**Table 1:** Baseline demographics of participants.

Demographics	Total, mean (SD) (N=2113)	Male, mean (SD) (n=860)	Female, mean (SD) (n=1253)	*P*
Age (years)	44.54 (10.72)	44.61 (10.98)	44.49 (10.54)	.81
Starting weight, (kg)	99.76 (22.92)	110.56 (22.43)	92.35 (20.14)	<.001
Starting BMI (kg/m^2^)	33.84 (6.80)	34.03 (6.35)	33.71 (7.09)	.27

**Table 2:** Weight-loss outcomes at 6 months.

Outcome measures	Total, mean (SE) (N=2113)	Male, mean (SE) (n=860)	Female, mean (SE) (n=1253)	*P*
Weight loss (%)	–5.58 (0.12)	–5.90 (0.12)	–5.36 (0.12)	.02
BMI change	–1.91 (0.04)	–2.04 (0.07)	–1.82 (0.05)	.01
Lost 5% of baseline weight (%)	51.87 (0.01)	54.30 (0.02)	50.20 (0.01)	.07

**Table 3:** Multiple regression models identifying predictors of weight loss at 6 months.

Models		Coefficients				Model summary		
		Beta (SE)	*t* (df)	*P*	*R*^2^		Adjusted *R*^2^	*P*
**Self-weigh-in**					.103		.102	<.001
	Weigh-ins/week (n)	–1.25 (0.19)	–6.54 (2110)	<.001				
	Weeks with ≥3 weigh-ins (%)	0.018 (0.01)	1.59 (2110)	.11				
**Activity**					.152		.150	<.001
	Tracker days/week	–0.54 (0.10)	–5.419 (2107)	<.001				
	Steps/day	–0.0002 (0.0001)	–1.863 (2107)	.06				
	Highly active minutes/day	–0.06 (0.01)	–4.288 (2107)	<.001				
	Fairly active minutes/day	0.003 (0.004)	0.693 (2107)	.49				
	Lightly active minutes/day	–0.002 (0.003)	–0.818 (2107)	.41				
**Nutrition/food logging**					.123		.121	<.001
	Food logs/week (n)	0.01 (0.04)	0.245 (2108)	.81				
	Food log days/week (n)	–1.92 (0.20)	–9.362 (2108)	<.001				
	Weeks with ≥5 logs (%)	0.08 (0.01)	5.935 (2108)	<.001				
	Weeks with ≥15 logs (%)	–0.01 (0.01)	–0.654 (2108)	.51				
**Overall**					.197		.194	<.001
	Weigh-ins/week (n)	–0.417 (0.07)	–5.619 (2106)	<.001				
	Tracker days/week (n)	–0.112 (0.10)	–1.081 (2106)	.28				
	Steps/day (n)	–0.0001 (0.00006)	–2.269 (2106)	.02				
	Highly active mins/day (n)	–0.05 (0.01)	–4.420 (2106)	<.001				
	Food log days/week (n)	–1.30 (0.19)	–6.777 (2106)	<.001				
	Weeks with ≥5 logs (%)	0.06 (0.01)	5.097 (2106)	<.001				

### Identifying Behaviors That Matter

#### Model for Self-Weigh-In Behavior as a Predictor of Weight Change

To identify important self-weigh-in measures for predicting weight change, a regression model was built (*R*^2^=.103, *P*<.001) containing the number of weigh-ins per week and the percentage of weeks with three or more weigh-ins. Table 3 shows that only the number of weigh-ins per week was identified as a significant predictor of weight change (*P*<.001).

#### Model for Activity-Related Behaviors as Predictor of Weight Change

To identify significant activity-related measures, a multiple regression model was constructed (*R*^2^=.152, *P*<.001) containing the number of activity tracker usage days per week, the number of steps per day, and the number of highly, fairly, and lightly active minutes per day. Table 3 displays that the number of activity tracker days per week (*P*<.001) and the number of highly active minutes per day (*P*<.001) were significant predictors of weight change. Though the number of steps per day was not significant (*P*=.06), it was selected to be included as a predictor for weight change in the overall model based on previous study indications [27,30,39].

#### Model for Food Logging-Related Behaviors as a Predictor of Weight Change

To identify significant nutrition/food logging-related measures, a multiple regression model was constructed (*R*^2^=.123, *P*<.001) containing the number of food logs per week, the number of food log days per week, the percentage of weeks with five or more food logs, and the percentage of weeks with 15 or more food logs. Table 3 shows that the number of food log days and the percentage of weeks with five or more food logs were significant predictors of weight change.

#### Overall Multiple Regression Model

An overall regression model was built to predict weight change at 6 months by including all significant predictors from the self-weigh-in, activity, and food logging model. This multiple regression model (*R*^2^=.197, *P*<.001) included the number of weigh-ins per week, the number of tracker usage days per week, the number of steps per week, the number of highly active minutes per week, the number of food log days per week, and the percentage of weeks with five or more food logs. Except the tracker usage days per week, all other behaviors/measures were found to be significant predictors of weight change, as shown in Table 3.

To further verify the significance of the selected behaviors/measures, an “all possible regressions” method was used to derive the best-fitting overall model. This approach of model selection determined the final model by performing an exhaustive search for the best subsets of the 11 measures listed under the primary regression models for predicting weight loss. All possible regressions included only the main effects; interactions were beyond the scope of this analysis. The best regression model contained the same five significant predictors of the overall model reported in Table 3. The next section focuses on analyzing the five significant predictors.

### The Five Significant Predictors of Weight Loss

#### Self-Weigh-In

Based on the self-weigh-in data from 0 to 6 months, a higher weigh-in frequency was significantly associated with a higher level of weight loss at 6 months. Clinically significant weight loss (5%) was associated with at least three weigh-ins per week (see Table 4). The results of one-way ANOVA showed a significant difference of mean weight loss between different weigh-in levels (*P*<.001). A subsequent Tukey test confirmed the significant differences between the “≥5” weigh-in level and the remaining three levels (*P*<.001 for all) and between weigh-in levels “3 to 4” and “1 to 2” (*P*=.002) and between weigh-in levels “3 to 4” and “<1” (*P*=.02). Similar ANOVA tests were performed on male and female participants separately and a significant difference in mean weight loss between different weigh-in levels was found (male: *P*<.001; female: *P*<.001; see Multimedia Appendix 4, Table S2).

**Figure 2:**
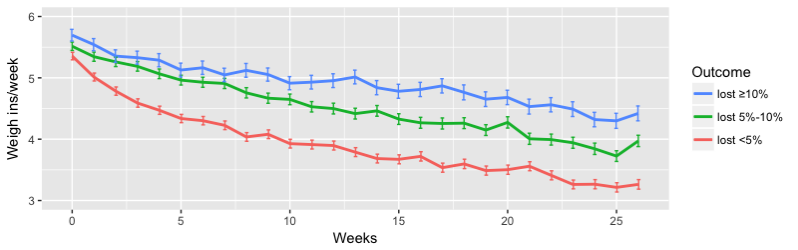
Weekly mean weigh-in frequency of participants between 0 and 6 months. Error bars indicate the standard error of the mean.

The analysis of self-weigh-in frequency of participants with different levels of weight loss showed that a higher weigh-in frequency was significantly associated with groups with higher levels of weight loss. Figure 2 presents the mean weekly weigh-in frequency among participants of three outcome levels: “lost ≥10%” (388/2113, 18.36%), “lost 5%-10%” (707/2113, 34.46%), and “lost <5%” (1018/2113, 48.18%). For all other analyses throughout the paper, behavior frequency based on outcome levels uses the same outcome-based participant groups. It showed a clear difference in weigh-in frequency throughout the 6-month program. The mean weigh-in frequencies over 6 months were mean 4.70 (SE 0.09), mean 4.21 (SE 0.07), and mean 3.40 (SE 0.05) weigh-ins per week for the lost ≥10%, lost 5%-10%, and lost <5% groups, respectively (*P*<.001). Additional ANOVA tests performed on male and female participants separately showed a similar significant difference of mean weigh-in frequency between different outcome levels (male: *P*<.001; female: *P*<.001, see Multimedia Appendix 4, Table S3).

**Table 4:** Weight-loss outcomes of participants for different behavior frequencies.

Self-monitoring behaviors		n (%)		Weight loss (%), mean (SE)		*P*	
**Weigh-in frequency per week**						<.001	
	<1		89 (4.21)		–3.41 (0.58)		
	1-2		636 (30.10)		–4.08 (0.20)		
	3-4		690 (33.65)		–5.09 (0.19)		
	≥5		698 (33.03)		–7.82 (0.20)		
**Steps per day**						<.001	
	<5000		797 (37.72)		–3.68 (0.17)		
	5000-7499		604 (28.58)		–5.56 (0.20)		
	7500-9999		429 (20.30)		–7.03 (0.26)		
	≥10,000		283 (13.39)		–9.03 (0.34)		
**Highly active minutes per week**						<.001	
	<60		897 (42.41)		–4.14 (0.17)		
	60-119		525 (24.82)		–5.71 (0.21)		
	120-179		299 (14.14)		–5.85 (0.29)		
	≥180		394 (18.63)		–8.64 (0.28)		
**Food log days per week**						<.001	
	<1		316 (14.96)		–3.67 (0.33)		
	1-2		596 (28.21)		–4.32 (0.20)		
	3-4		565 (26.74)		–5.15 (0.19)		
	≥5		636 (30.10)		–8.20 (0.21)		
**Food logs per week**						<.001	
	<5		617 (29.20)		–4.37 (0.21)		
	5-9		405 (19.17)		–4.66 (0.24)		
	10-14		297 (14.06)		–5.11 (0.29)		
	15-19		247 (11.69)		–5.46 (0.32)		
	≥20		547 (25.89)		–8.10 (0.23)		

#### Daily Steps

Based on the steps data of participants between 0 and 6 months, a higher level of daily steps was significantly associated with a higher level of weight loss. Table 4 presents the results of the weight-loss analysis performed by dividing the participants to different levels of daily step counts. Daily steps of 5000 to 7499 or more were associated with clinically significant weight loss at 6 months. One-way ANOVA showed a significant difference in mean weight loss between different daily step levels (*P*<.001). A subsequent Tukey test confirmed significant mean differences between all levels of daily steps (*P*<.001). Similar ANOVA tests performed on male and female participants separately showed a significant difference in mean weight loss between the different daily step levels (male: *P*<.001; female: *P*<.001; see Multimedia Appendix 4, Table S4).

**Figure 3:**
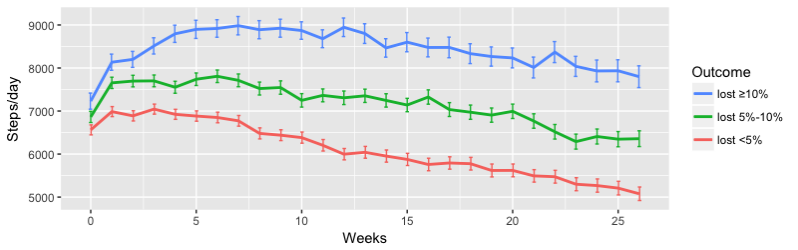
Weekly mean steps per day of participants between 0 and 6 months. Error bars indicate the standard error of the mean.

The analysis of daily steps of participants with different levels of weight loss showed that a higher daily step count was significantly associated with groups with higher levels of weight loss. Figure 3 presents the weekly mean steps per day among participants of three outcome levels. The lost ≥10% group consistently maintained significantly higher daily steps throughout the 6-month program. The mean daily steps over 6 months were mean 8077.79 (SE 171.52), mean 6657.09 (SE 117.13), and mean 5276.91 (SE 95.08) steps per day for the lost ≥10%, lost 5%-10%, and lost <5% groups, respectively (*P*<.001). Male and female participants separately showed a similar significant difference in mean daily steps between the different outcome levels (male: *P*<.001; female: *P*<.001; see Multimedia Appendix 4, Table S5).

#### Highly Active Minutes

Higher levels of highly active minutes were significantly associated with higher levels of weight loss. Percentage of weight loss was calculated by dividing the participants into different levels of highly active minutes per week (Table 4). Higher-intensity activity for 60 minutes or more per week was associated with clinically significant weight loss. There was a significant difference in mean weight loss between different weekly active minutes levels (*P*<.001). A subsequent Tukey test showed significant differences between the “≥180” active minutes level and the remaining three levels (*P*<.001 for all) and between “120-179” active minutes level and “<60” (*P*<.001) and between “60-119” active minutes level and “<60” (*P*<.001). Similar ANOVA tests performed on male and female participants separately showed a similar significant difference in mean weight loss between different daily highly active minutes levels (male: *P*<.001; female: *P*<.001, see Multimedia Appendix 4, Table S6).

**Figure 4:**
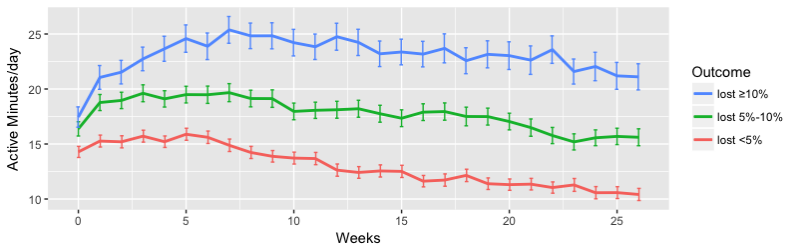
Weekly mean highly active minutes of participants between 0 and 6 months. Error bars indicate the standard error of the mean.

The analysis of highly active minutes of the participants with different levels of weight loss showed that higher amounts of highly active minutes were significantly associated with groups with higher levels of weight loss. Figure 4 shows the weekly mean highly active minutes among participants in three outcome levels. Similar to daily steps, the lost ≥10% group consistently had a significantly higher level of high-intensity activity throughout the 6-month program. The mean weekly highly active minutes over 6 months were mean 154.33 (SE 6.47), mean 115.63 (SE 3.91), and mean 79.03 (SE 2.53) minutes per week for the lost ≥10%, lost 5%-10%, and lost <5% groups, respectively (*P*<.001). Male and female participants separately showed a similar significant difference in mean highly active minutes between different outcome levels (male: *P*<.001; female: *P*<.001; see Multimedia Appendix 4, Table S7).

#### Food Log Days

Analysis of participants’ food log data over 0 to 6 months showed that a higher number of food log days per week was significantly associated with a higher level of weight loss. Table 4 presents the weight-loss percentages of the groups of participants at different levels of food log days per week. Clinically significant weight loss was associated with at least three or more days of food logging per week. There was a significant difference in weight loss between different levels of weekly food log days (*P*<.001). A subsequent Tukey test found significant differences between “≥5” food log days and the remaining three levels (*P*<.001 for all) and between “3-4” and “1-2” food log days (*P*=.03) and between “3-4” and “<1” food log days (*P*<.001). Similar ANOVA tests performed on male and female participants separately showed a similar significant difference in mean weight loss between different numbers of food log days (male: *P*<.001; female: *P*<.001; see Multimedia Appendix 4, Table S8).

**Figure 5:**
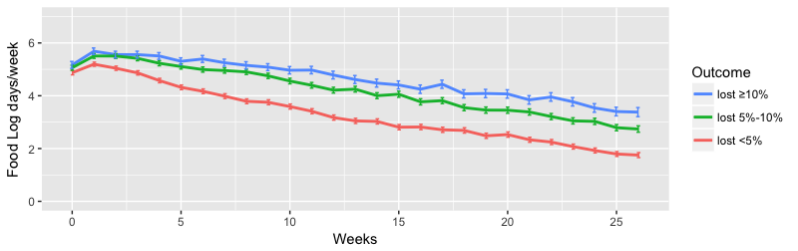
Weekly mean food log days of participants between 0 and 6 months. Error bars indicate the standard error of the mean.

The analysis of food log days of the participants with different levels of weight loss showed that a higher number of food log days per week was significantly associated with groups with higher levels of weight loss. Figure 5 shows the weekly mean food log days among the participants in the three outcome levels. Participants in the higher outcome levels logged their food a significantly higher number of days throughout the 6-month program than the lowest outcome group. The mean weekly food log days over 6 months were mean 4.44 (SE 0.11), mean 3.92 (SE 0.08), and mean 2.90 (SE 0.60) days per week for the lost ≥10%, lost 5%-10%, and lost <5% groups, respectively (*P*<.001). Male and female participants separately showed a similar significant difference in mean food log days between different outcome levels (male: *P*<.001; female: *P*<.001; see Multimedia Appendix 4, Table S9).

#### Percentage of Weeks With Five or More Food Logs

Additional analysis of food logs showed that participants with a higher level of weight loss were significantly associated with a higher percentage of weeks with five or more food logs. There was a significant difference in percentage of weeks with five or more food logs between different weight loss outcome levels (lost ≥10%: mean 69.40%, SE 1.72; lost 5%-10%: mean 63.61%, SE 1.20; lost <5%: mean 49.14%, SE 0.97; *P*<.001). A subsequent Tukey test was performed, which found significant mean differences between all outcome levels (lost ≥10% and lost <5%: *P*<.001; lost ≥10% and lost 5%-10%: *P*=.01; lost 5%-10% and lost <5%: *P*<.001). Additional ANOVA tests performed on male and female participants separately showed a similar significant difference in percentage of weeks with five or more food logs between the different outcome levels (male: *P*<.001; female: *P*<.001; see Multimedia Appendix 4, Table S10).

Based on the analysis presented in previous sections, food logging is very critical for weight loss during the 6-month intervention. Hence, additional analysis is presented in Table 4 that shows the percentage weight loss for participants in different food log groups per week. A higher number of food logs per week was significantly associated with a higher level of weight loss (*P*<.001). Further analysis to understand differences in weight-loss outcomes for male and female participants between different food log groups showed a similar difference (male: *P*<.001; female: *P*<.001; see Multimedia Appendix 4, Table S11).

## Discussion

### Principal Findings

The results provide strong support for the use of self-monitoring in weight-management programs. Participants who complied more with body weight, physical activity, and food intake self-monitoring lost more weight than those who complied less. In a multiple regression equation, each category of self-monitoring contributed significantly to the prediction of weight loss. Furthermore, the independent analysis showed a significant association between each self-monitoring behavior and weight loss. Overall, the use of self-monitoring was found to have a high impact on weight management.

Advances in technology, such as wireless scales and physical activity trackers, make it easier to self-monitor weight and physical activity, and are recommended in weight-management programs. Food logging still requires that a participant take time to record food intake, but technology has made it a faster and simpler process. However, there is a great need for developing new technology to reduce the time, effort, and accuracy in self-monitoring food intake.

Some report that self-monitoring of single behaviors, such as body weight and physical activity [2,27,40], may not be associated with greater weight loss. Our results found benefits of self-monitoring in each behavior category: weight, physical activity, and food intake. We found that self-monitoring all these behaviors together had the greatest predictive value for weight loss. Based on these results, it may be important to promote these self-monitoring behaviors together in an intervention or weight-loss program.

### Significant Predictors of Weight Loss

#### Self-Weigh-In

The number of self-weigh-ins per week was identified as a significant predictor of weight loss (*P*<.001). Self-weighing at least three times per week is associated with higher weight loss. An even higher level of weight loss is associated when weighing more than five times per week. Evidence has shown that instructing participants to weigh in at least three times per week does increase weight loss during an active weight-loss period, and a higher frequency of weighing in is associated with greater weight-loss success [15-18]. Additionally, females tend to weigh in more frequently than males, which is a unique finding due to an historically small percentage of male participants in weight-management studies and a lack of evidence around gender comparison [15-18,20,25,40-48].

#### Daily Steps

The number of steps per week (*P*=.02) is a significant predictor of weight change. Higher step counts are associated with greater levels of weight loss, which has been shown in previous literature [27,30,39]. Also, our results confirm that men tend to have a higher daily step count than women, similar to that seen in the literature [26].

#### Highly Active Minutes

A minimum of 60 highly active minutes per week is significantly associated with higher levels of weight loss. Greater levels of highly active minutes are also significantly associated with higher weight loss outcomes. Males overall have a significantly higher level of active minutes than females at all weight-loss outcome levels (*P*<.001). Currently, there is a lack of evidence around measuring active minutes with activity trackers associated with weight-loss outcomes. However, there is some evidence that men log more exercise than females and have a greater exercise dependence [33,43,49].

#### Food Log Days

A higher number of food log days per week increases adherence to the self-monitoring behavior of food logging, which supports behavioral change as explained through self-regulation theory [31]. Food logging at least three days per week was significantly associated with higher levels of weight loss. Other studies have found that greater weight loss is achieved with a higher frequency of food logs, specifically three or more days per week [31-33,36,43].

#### Percentage of Weeks With Five or More Food Logs

A higher percentage of weeks with five or more food logs is significantly associated with higher levels of weight loss (*P*<.001). Additionally, the more times a participant logs their food per week increases their likelihood of successful weight loss [31-33]. Women tend to log their food more frequently than men do. However, this is a unique finding due to a historically small percentage of male participants in weight-management studies and a lack of evidence around gender comparison [31-35].

### Strengths and Limitations

This study has several strengths, including the reporting of real-world weight-loss outcomes and providing a more focused analysis into weight-management behaviors to determine what behaviors are more significant in a behavioral weight-loss program. Participants were clients of Retrofit and not recruited or provided with incentives to participate in the study. All clients who met the starting BMI, age, and weight inclusion criteria and logged a weight at 6 months were included as participants and not removed from the population due to lack of success on the program, which is an uncommon research practice [44]. We conclude that this study adds value and brings a novel approach to the best practices around behaviors in weight management. Additionally, gender comparisons were able to be reported due to the unusually high population of males enrolled as participants, which is also a significant strength to understand which behaviors are more valuable to men and women in the weight-loss process.

The study also has some limitations, including the retrospective analysis study design, which does not allow any causal inferences based on the critical observations. Moreover, the adherence to different behaviors were evaluated using data from program completers, which limits the ability to generalize impact of the behavior on all participants. However, to determine effective levels of self-monitoring that can guarantee clinically significant weight loss, it is critical to study participants with known end weights. Lastly, due to use of the real-world population in this study, it is unknown if participants were integrating any other self-monitoring devices or practices outside of the Retrofit Program components.

### Future Research

With a lack of real-world research in the commercial weight-loss industry, Retrofit encourages all commercial weight-loss programs to publish similar data to enhance understanding of which self-monitoring behaviors matter most in a weight-loss program. Reporting real-world data in relation to targeted behaviors allows commercial weight-loss programs to not only structure protocols and client strategies to increase weight-loss success, but also improve a participant’s weight maintenance success. By narrowing in on the specific behaviors to build as life-long habits, commercial weight-loss programs will increase efficacy and establish our ability as an industry to overcome the obesity crisis.

Recommended future research includes studying self-monitoring behaviors beyond 6 months and each behavior’s impact on weight maintenance. Also, further analysis around gender differences and self-monitoring behaviors is of interest to determine if specific behaviors should be encouraged more frequently among female versus male participants, specifically around food logging and activity levels.

### Conclusions

In conclusion, participants on the Retrofit Program lost a mean –5.58% (SE 0.12) and had a BMI change of mean –1.91 (SE 0.04) in 6 months with nearly 51.87% (1096/2113) of participants losing 5% or more of their baseline weight. Self-monitoring behaviors, such as self-weigh-in, daily step counts, high-intensity activity, and persistent food logging were shown to be significant predictors of weight change at 6 months. Specifically, weighing in three times or more per week, having a minimum of 60 highly active minutes per week, food logging for three days or more per week, and having a higher percentage of weeks with five or more food logs increased participant’s weight-loss success.

## Appendix

Multimedia Appendix 1 Retrofit logo.

Multimedia Appendix 2 The technology provided included a Wi-Fi–enabled scale, activity tracker, and access to a private dashboard. The dashboard was accessible via Web and mobile apps.

Multimedia Appendix 3 Retrofit client dashboard for logging food and exercise accessible via Web and mobile apps.

Multimedia Appendix 4 Supplementary Tables.

## Abbreviations

BMI: body mass index
