# Supplementary figures and images for: What Matters in Weight Loss? An In-Depth Analysis of Self-Monitoring
Source: J Med Internet Res. 2017 May 12;19(5):e160. doi: 10.2196/jmir.7457 (PMC5446667; doi:10.2196/jmir.7457)

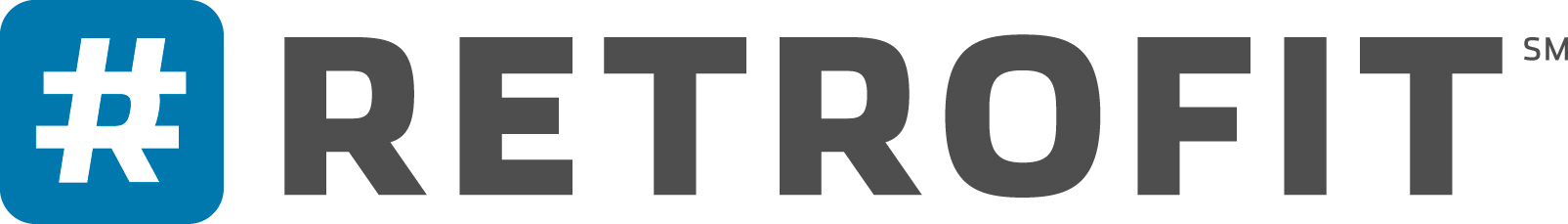

Supplement: Multimedia Appendix 1 [file jmir_v19i5e160_app1.jpg]

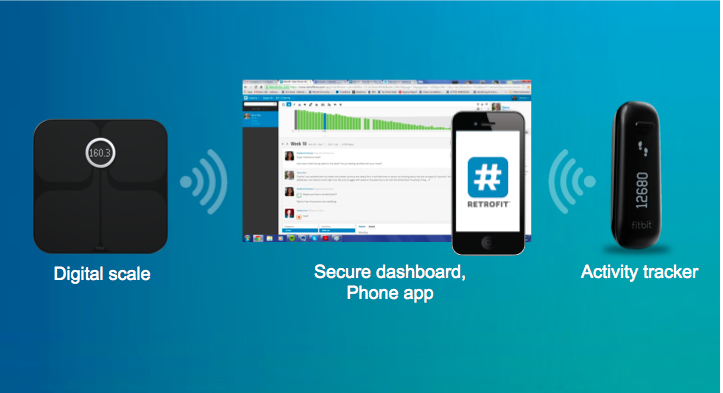

Supplement: Multimedia Appendix 2 [file jmir_v19i5e160_app2.png]

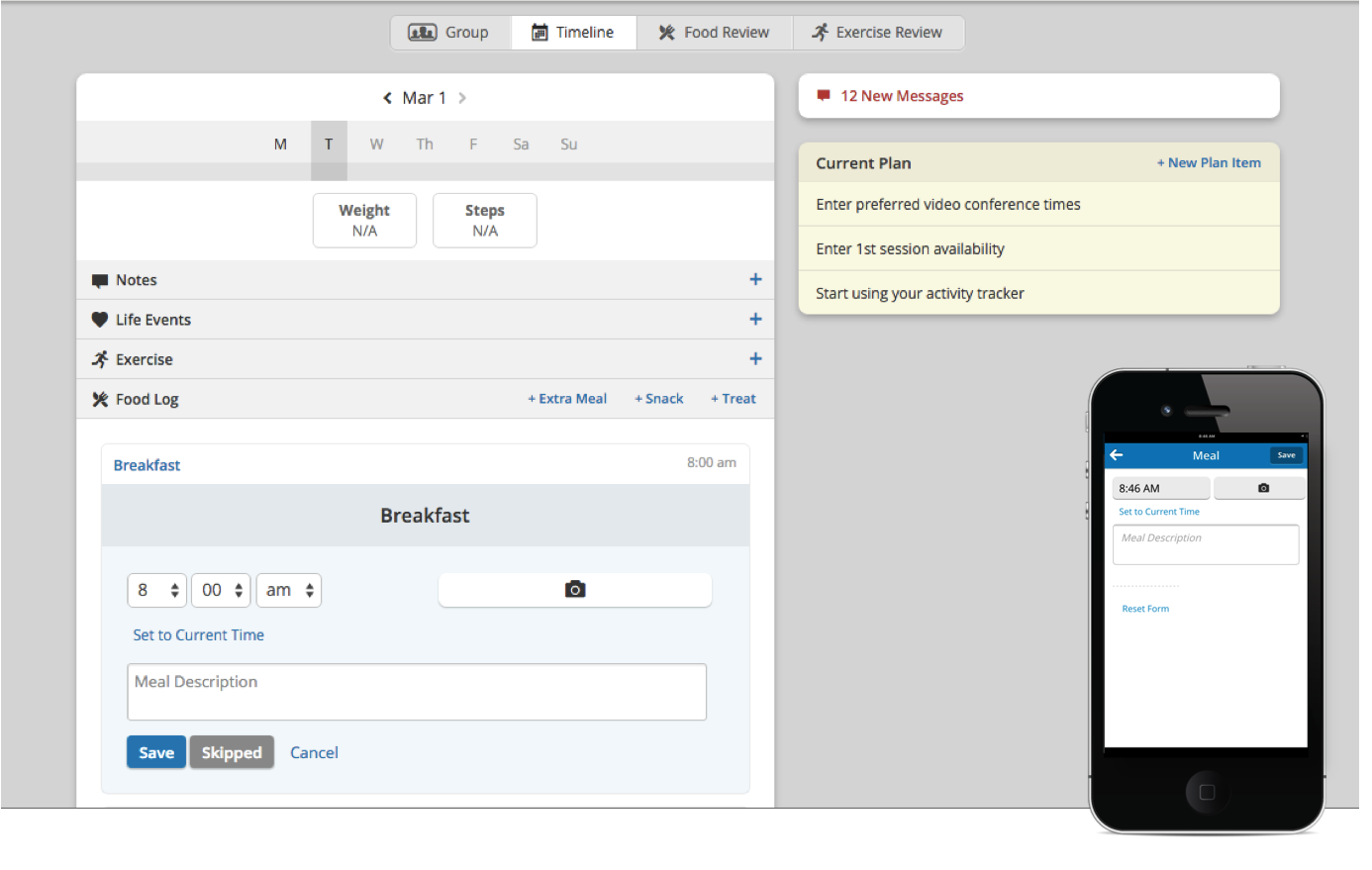

Supplement: Multimedia Appendix 3 [file jmir_v19i5e160_app3.png]
