# Supplementary material for: What Matters in Weight Loss? An In-Depth Analysis of Self-Monitoring
Source: J Med Internet Res. 2017 May 12;19(5):e160. doi: 10.2196/jmir.7457 (PMC5446667; doi:10.2196/jmir.7457)
Supplement: Multimedia Appendix 4 [file jmir_v19i5e160_app4.pdf]

Supplementary Tables

Table S1. Baseline demographics

**Demographics**	**All Participants** Mean (SD) (n = 3116)	**Completers** Mean (SD) (n=2113, 41%)	**Non-completers** Mean (SD) (n=1253, 59%)	***P-value***
Age, years	43.70 (10.77)	44.54 (10.72)	42.01 (10.69)	**< 0.001**
Starting Weight, kilograms	99.25 (22.73)	99.76 (22.92)	98.22 (22.32)	0.071
Starting BMI, kg/m^2	33.79 (6.72)	33.84 (6.80)	33.69 (6.56)	0.551

Table S2. Weight-loss outcomes of participants with different weekly weigh-in frequency

**Weigh-in Frequency per week**	**Overall**		**Female**		**Male**		*p-value*
	N (%)	Weight Loss % Mean (SE)	N (%)	Weight Loss % Mean (SE)	N (%)	Weight Loss % Mean (SE)	
Less than 1	89 (4%)	-3.41 (0.58)	46 (4%)	-3.87% (0.77)	43 (5%)	-2.93% (0.87)	0.420
1 to 3	636 (30%)	-4.08 (0.20)	375 (30%)	-3.82% (0.26)	261 (30%)	-4.44% (0.31)	0.126
3 to 5	690 (33%)	-5.09 (0.19)	392 (31%)	-4.75% (0.26)	298 (35%)	-5.52% (0.28)	**0.042**
5 or more	698 (33%)	-7.82 (0.20)	440 (35%)	-7.53% (0.25)	258 (30%)	-8.30% (0.34)	0.066
p-value		**< 0.0001**		**< 0.0001**		**< 0.0001**	

Table S3. Weigh-in frequency of participants with different levels of outcome at 6 months

**Outcome Levels**	**Overall**		**Female**		**Male**		*p-value*
	N (%)	Weigh-in/Week Mean (SE)	N (%)	Weigh-in/Week Mean (SE)	N (%)	Weigh-in/Week Mean (SE)	
Loss ≥ 10%	388 (18%)	4.70 (0.09)	220 (18%)	4.89 (0.12)	168 (20%)	4.44 (0.14)	**0.013**
Loss 5-10%	707 (34%)	4.21 (0.07)	406 (32%)	4.22 (0.09)	301 (35%)	4.18 (0.10)	0.741
Loss < 5%	1018 (48%)	3.40 (0.05)	628 (50%)	3.53 (0.07)	392 (45%)	3.19 (0.08)	**0.001**
p-value		**< 0.0001**		**< 0.0001**		**< 0.0001**	

Table S4. Weight-loss outcomes of participants with different daily step counts

**Steps per Day**	**Overall**		**Female**		**Male**		*p-value*
	N (%)	Weight Loss % Mean (SE)	N (%)	Weight Loss % Mean (SE)	N (%)	Weight Loss % Mean (SE)	
Less than 5K	797 (38%)	-3.68% (0.17)	494 (40%)	-3.68% (0.22)	303 (35%)	-3.67% (0.27)	0.977
5K to 7.5K	604 (29%)	-5.56% (0.20)	375 (30%)	-5.45% (0.25)	229 (27%)	-5.73% (0.32)	0.487
7.5K to 10K	429 (20%)	-7.03% (0.26)	242 (19%)	-6.94% (0.36)	187 (22%)	-7.15% (0.36)	0.685
10K or more	283 (13%)	-9.03% (0.34)	142 (11%)	-8.76% (0.47)	141 (16%)	-9.31% (0.49)	0.417
p-value		**< 0.0001**		**< 0.0001**		**< 0.0001**	

Table S5. Daily step counts of clients with different levels of outcome at 6 months

**Outcome Levels**	**Overall**		**Female**		**Male**		*p-value*
	N (%)	Steps/Day Mean (SE)	N (%)	Steps/Day Mean (SE)	N (%)	Weigh-in/Week Mean (SE)	
Loss ≥ 10%	388 (18%)	8077 (171.52)	220 (18%)	7839 (214)	168 (20%)	8390 (279)	0.119
Loss 5-10%	707 (34%)	6657 (117.13)	406 (32%)	6390 (148)	301 (35%)	7017 (187)	**0.009**
Loss < 5%	1018 (48%)	5277 (95.08)	628 (50%)	5240 (114)	392 (45%)	5335 (168)	0.639
p-value		**< 0.0001**		**< 0.0001**		**< 0.0001**	

Table S6. Weight-loss outcomes of participants with different Highly active minutes per week

Highly Active Min. Per Week	Overall		Female		Male		*p-value*
	N (%)	Weight Loss % Mean (SE)	N (%)	Weight Loss % Mean (SE)	N (%)	Weight Loss % Mean (SE)	
Less than 60	897 (42%)	-4.14 (0.17)	624 (50%)	-4.20% (0.21)	273 (32%)	-4.01% (0.32)	0.614
60 to 120	525 (25%)	-5.71 (0.21)	334 (27%)	-5.74% (0.26)	191 (22%)	-5.65% (0.33)	0.832
120 to 180	299 (14%)	-5.85 (0.29)	158 (12%)	-6.33% (0.42)	141 (16%)	-5.32% (0.40)	0.81
180 or more	394 (19%)	-8.64 (0.28)	138 (11%)	-9.04% (0.50)	256 (30%)	-8.42% (0.34)	0.308
p-value		**< 0.0001**		**< 0.0001**		**< 0.0001**	

Table S7. Highly active minutes per week of clients with different levels of outcome at 6 months

Outcome Levels	Overall		Female		Male		*p-value*
	N (%)	Highly Active Mins/Week Mean (SE)	N (%)	Highly Active Mins/Week Mean (SE)	N (%)	Highly Active Mins/Week Mean (SE)	
Loss ≥ 10%	388 (18%)	154 (6.47)	220 (18%)	126 (7.48)	168 (20%)	192 (10.63)	**< 0.0001**
Loss 5-10%	707 (34%)	116 (3.91)	406 (32%)	90 (4.03)	301 (35%)	151 (6.92)	**< 0.0001**
Loss < 5%	1018 (48%)	79 (2.53)	628 (50%)	65 (2.50)	392 (45%)	102 (4.99)	**< 0.0001**
p-value		**< 0.0001**		**< 0.0001**		**< 0.0001**	

Table S8. Weight-loss outcomes of participants with different number of food log days per week

Food Log Days Per Week	Overall		Female		Male		*p-value*
	N (%)	Weight Loss % Mean (SE)	N (%)	Weight Loss % Mean (SE)	N (%)	Weight Loss % Mean (SE)	
Less than 1	316 (15%)	-3.67 (0.33)	146 (12%)	-3.13 (0.50)	170 (20%)	-4.14 (0.44)	0.129
1 to 3	596 (28%)	-4.32 (0.20)	357 (28%)	-4.11 (0.26)	239 (28%)	-4.64 (0.30)	0.188
3 to 5	565 (27%)	-5.15 (0.19)	363 (29%)	-4.99 (0.25)	202 (23%)	-5.45 (0.31)	0.249
5 or more	636 (30%)	-8.20 (0.21)	387 (31%)	-7.88 (0.27)	249 (29%)	-8.68 (0.34)	0.065
p-value		**< 0.0001**		**< 0.0001**		**< 0.0001**	

Table S9. Food log days per week of clients with different levels of outcome at 6 months

Outcome Levels	Overall		Female		Male		*p-value*
	N (%)	Food Log Days/Week Mean (SE)	N (%)	Food Log Days/Week Mean (SE)	N (%)	Food Log Days/Week Mean (SE)	
Loss ≥ 10%	388 (18%)	4.44 (0.11)	220 (18%)	4.64 (0.14)	168 (20%)	4.18 (0.19)	0.051
Loss 5-10%	707 (34%)	3.92 (0.08)	406 (32%)	4.01 (0.10)	301 (35%)	3.80 (0.13)	0.182
Loss < 5%	1018 (48%)	2.90 (0.60)	628 (50%)	3.08 (0.07)	392 (45%)	2.62 (0.10)	**0.0003**
p-value		**< 0.0001**		**< 0.0001**		**< 0.0001**	

Table S10. Percentage of five food log weeks in clients with different levels of outcome at 6 months

**Outcome Levels**	**Overall**		**Female**		**Male**		*p-value*
	N (%)	% of 5x Food-log Weeks Mean (SE)	N (%)	% of 5x Food-log Weeks Mean (SE)	N (%)	% of 5x Food-log Weeks Mean (SE)	
Loss ≥ 10%	388 (18%)	69.40 (1.72)	220 (18%)	72.55 (2.13)	168 (20%)	65.26 (2.81)	**0.039**
Loss 5-10%	707 (34%)	63.61 (1.20)	406 (32%)	65.37 (1.52)	301 (35%)	61.25 (1.93)	0.094
Loss < 5%	1018 (48%)	49.14 (0.97)	628 (50%)	51.91 (1.19)	392 (45%)	44.70 (1.61)	**0.0003**
p-value		**< 0.0001**		**< 0.0001**		**< 0.0001**	

Table S11. Weight-loss outcomes of participants with different number of food logs per week

Food Logs Per Week	Overall		Female		Male		*p-value*
	N (%)	Weight Loss % Mean (SE)	N (%)	Weight Loss % Mean (SE)	N (%)	Weight Loss % Mean (SE)	
Less than 5	617 (29%)	-4.37 (0.21)	322 (26%)	-4.20 (0.31)	295 (35%)	-4.55 (0.30)	0.415
5 to 10	405 (19%)	-4.66 (0.24)	249 (20%)	-4.42 (0.31)	156 (18%)	-5.04 (0.39)	0.209
10 to 15	297 (14%)	-5.11 (0.29)	191 (15%)	-5.02 (0.38)	106 (12%)	-5.28 (0.46)	0.654
15 to 20	247 (12%)	-5.46 (0.32)	150 (12%)	-4.85 (0.39)	97 (11%)	-6.41 (0.54)	**0.020**
20 or more	547 (26%)	-8.10 (0.23)	341 (27%)	-7.77 (0.29)	206 (24%)	-8.56 (0.38)	0.097
p-value		**< 0.0001**		**< 0.0001**		**< 0.0001**	
